# DNA Phase Transition in Charge Neutralization and Comformation Induced by Trivalent-Hydrolysed Metal Ions

**DOI:** 10.3390/polym10040394

**Published:** 2018-04-02

**Authors:** Zhaoxu Luo, Yanwei Wang, Shuhang Li, Guangcan Yang

**Affiliations:** College of Mathematical, Physics and Electronic Information Engineering, Wenzhou University, Wenzhou 325035, China; 13216018553@163.com (Z.L.); wangyw@wzu.edu.cn (Y.W.); wzulsh@163.com (S.L.)

**Keywords:** DNA Phase Transition, hydrolysed metal ions, charge neutralization, AFM

## Abstract

It is well known that common trivalent counter ions can induce DNA compaction or condensation but are unable to invert DNA surface charge in a normal aqueous solution. In the present study, we found that trivalent-hydrolysed metal ions (Fe^3+^, Al^3+^) are not only capable of inducing DNA condensation, but they also invert the electrophoretic mobility of DNA by electrophoretic light scattering and single molecular techniques. In comparison with neutral trivalent cations, hydrolysed metal ions such as Fe^3+^ can induce DNA condensation at a much lower concentration of cations, and its corresponding morphology of condensed DNA was directly observed by atomic force microscopy (AFM). The condensing and unravelling forces of DNA condensates were measured by tethering DNA by magnetic tweezers (MT) measurements at various concentration of Fe^3+^ and Al^3+^. We found that a coil–globule transition of DNA by hydrolysed metal ions not only was observed in DNA-complex sizes, but also in the curve of electrophoretic mobility of DNA in solution. In contrast, the transition was not observed in the case of neutral trivalent cations such as La^3+^ and Co^3+^. We attribute the transition and charge inversion to the ion-specific interaction between hydrolysed metal ions and phosphates of DNA backbone.

## 1. Introduction

DNA is an important biological polyelectrolyte with a high density of negative charge, resulting in very strong Coulomb repulsion between the nucleic acid segments. However, in reality, DNA chains exist as very compact globules in both prokaryotic and eukaryotic living cells [1]. Thus, physicochemical studies of the large structural changes of long DNAs are not only important in molecular biology but are also helpful in developing new techniques such as DNA extraction and gene therapy, in which DNA is compacted and transfected into cells and tissues to treat some genetic-related diseases [2,3,4].

Many condensing agents, such as multivalent cations, which are basic proteins, are able to induce DNA compaction or condensation [5,6,7,8,9,10,11,12]. In general, cations with a valence of greater than 3 are required to form DNA condensate in aqueous solution at room temperature. To overcome the strong Coulombic repulsion between segments of DNA, most of its charge has to be neutralized by the opposite charged counterions in solution. In some conditions, DNA as a polyelectrolyte can attract more opposite charges than its own nominal charge so that its effective charge changes from negative to positive, implying that charge inversion occurs. DNA condensation and its charge inversion have been experimentally investigated by Dynamic Light Scattering (DLS) [13], Atomic Force Microscopy (AFM) [14,15,16,17], and some recent developed single molecular techniques such as Optical tweezers (OT) and Magnetic Tweezers (MT) [18,19,20].

The counterintuitive charge inversion cannot be described in the framework of a purely electrostatic mean-field theory; instead, it is related to ion correlation and/or specific adsorption of multivalent ions (or multivalent-ion complexes) to the charged surface [21,22]. In correlation mechanism, ion size and spatial correlations of screening ions play a significant role to form a 2-dimensional Wigner crystal on the charge surface to attract additional counterions that result in charge inversion and, in turn, necessarily accompany and influence counter-ion-induced like-charge attraction. On the other hand, the ionic specificity arises, because, in addition to long-range electrostatic interactions, short-range interactions, which are quantum mechanical in origin and highly specific to the ion and the interfacial charged group, are in play and need to be considered. In reality, both mechanisms might be in action simultaneously, more or less.

It is often observed in aqueous media that different ions of the same valence give rise to dramatically different phases or charge distributions [23,24,25,26,27]. For example, Fe(III) and La(III), both in oxidation state +3, can exist in aqueous solution as ions or as complexes. The two ions show their quite distinctive behavior in solutions, as it was found that La^3+^ provides a reasonably close representation of a “classical” ion, that is, one whose interaction is dominated by standard statistical mechanics, while Fe^3+^, with the same valence and very similar radius, provides an extreme case of specificity in which interactions with the hydrophilic groups are driven by the formation of reversible covalent bonds [28,29,30,31].

Free iron is present at low concentrations in biological fluid and plays an important role in many biochemical processes such as DNA damage induced by xenobiotic-derived electrophiles such as alkyldiazonium ions and alkyl radicals, and accelerates potentially harmful free radical reactions; thus, the structural analysis of iron bound DNA complexes has major biochemical importance, in view of the important role of iron in the oxidative stress and due to its strong affinity towards complexation with cell components, particularly those of nucleic acids and their nucleobases. Moreover, Fe cation is capable of binding to many biological targets including DNA and catalyzing a free radical reaction that will lead to a site-specific damage [32,33]. 

On the other hand, we focus on the effects of the trivalent metal ions Al^3+^ and Fe^3+^, since they are related to the effects of pH variations arising from the metal hydrolysis. We found that the common ions can induce DNA condensation but are unable to invert its charge. In contrast, site specific ions can not only condense DNA but also invert its charge. In the meanwhile, we found that individual DNAs undergo a marked discrete transition from an elongated coil into a collapsed globule with an increase in the Fe^3+^ and Al^3+^ concentration. On the other hand, there is no such discrete transition of DNA conformation and its charge state with the addition of Co^3+^ and La^3+^. The results have been analyzed theoretically in terms of the classical two-state model and cooperative phase transition model of Zimm–Bragg, indicating that the site specific binding of the counterions is the main reason of the discrete transition.

## 2. Experimental Procedures

### 2.1. Materials

Double strands λ-phage DNA (48502 bp) was purchased from New England Biolabs (Ipswich, MA, USA), and its initial concentration was 500 ng/μL. The final DNA concentration in AFM and DLS was 1 ng/μL. All buffers used in MT, DLS, and AFM were the TRIS (10 mM, pH = 7.2). Iron(III) chloride hexahydrate (FeCl_3_·6H_2_O), Aluminum(III) chloride hexahydrate (AlCl_3_·6H_2_O), Lanthanum(III) chloride heptahydrate (LaCl_3_·7H_2_O), Hexamine cobalt(III) chloride ([Co(NH_3_)_6_]Cl_3_), and hydroxylmethylaminoethane (TRIS) were purchased from Sigma (Sain Louis, MO, USA) at high-purity grade (>99%) and were used without further purification. Purified water was obtained from a Milli-Q system (Millipore, Billerica, MA, USA), and TRIS buffer (10 mM, pH = 7.2) was used as both stock solution and experimental buffer solution. The DNA molecule for tethering by magnetic tweezers must be bound at one end to an immobile support (the glass sidewalls) and at the other end to a magnetic bead [34]. Thus, one end of DNA was modified by digoxigenin, and the other end was modified with biotin to provide connections to the anti-digoxigenin-coated glass sidewall and avidin-coated magnetic beads, respectively. As described before [35], we used about 1 μL stock solution of magnetic beads coated with streptavidin (M-280, Dynal Biotech, Wirral, UK), which was gently mixed with 0.5 μL modified DNA for 30 min to form DNA-bead constructs in 200 μL buffer solution. Antidigoxygenin was purchased from Roche Diagnostics (Rotkreuz, Switzerland). Mica for AFM imaging was cut into approximately 1 cm^2^ square pieces, and their surfaces were always freshly cleaved before use. All chemical agents were used as received, and all measurements were repeated at least twice to obtain consistent results.

### 2.2. Electrophoretic Mobility and Size Measurement by Dynamic Light Scattering (DLS)

The electrophoresis-mobility measurements were carried out by using a dynamic light scattering device of Malvern Zetasizer nano ZS90 (Malvern Instruments Limited Company, Malvern, UK) equipped with the patented M3-PALS technique, in which a He–Ne gas laser (λ = 633 nm) was used. The light scattering was collected by an avalanche photodiode mounted on the goniometer arm in the perpendicular direction to the incident light. The DNA samples were diluted to a concentration of 1 ng/ μL in a buffer solution containing 10 mM Tris (pH = 7.2), and then different concentrations of Fe^3+^ were added. All measurements were carried out after 5 min incubation at room temperature. During the measurement, an 1 mL volume of DNA solution was used, and the sample cell was kept at 25 °C.

In particle-size measurements, the laser power is automatically attenuated in order to make the count rate from the sample within acceptable limits. Clear disposable capillary cells were used. In the preparation of sample, we mixed 200 μL of trivalent metal ions (Fe^3+^, Al^3+^, La^3+^, [Co(NH_3_)_6_]^3+^) at various concentrations and 0.2 μL DNA (500 ng/μL) in a rotary mixer for 2 h rotating to make sure the system reached a thermodynamically stable state. The final DNA concentration is 1 ng/μL. Then, 120 μL sample solution was pipetted into a clear disposable capillary cell for particle size measurement. As in the measurement of electrophoretic mobility, the sample cell was also maintained at 25 °C. 

### 2.3. AFM Imaging and Magnetic Tweezers Tethering

The sample preparing procedure can briefly be described as follows: Mica disks of diameter 1 cm attached to glass slide were used as substrates for DNA adsorption. For each sample, the final concentration of DNA was 1 ng/μL and a drop of about 25 μL of Fe^3+^ mixture was deposited for 3 min on a freshly cleaved mica surface. The surface was rinsed with distilled water and dried with a gentle flow of nitrogen gas. 

The prepared samples were scanned by AFM (JPK Nano WizardIII, Berlin, Germany) in AC mode. A 125 μm long and 30 μm wide and 4 μm thickness silicon AFM probe with aluminum coating, spring constant 42 N/m, and resonance frequency of 320 kHz (NCHR-50, NanoWorld Corporation, Tokyo, Japan) was used. All images were captured from a 5 μm × 5 μm viewing area on the sample by a scan rate of 1.0 Hz. Each image was 512 × 512 pixels (4–6 nm/pixel). For each sample, 3–10 images were acquired from different regions within it.

**Figure 1:**
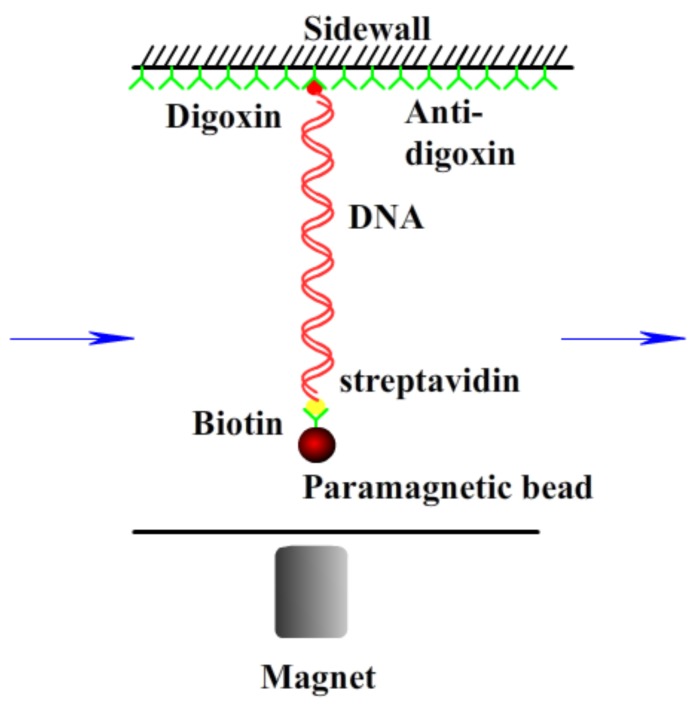
A schematic diagram of magnetic tweezers.

A transverse MT system (Figure 1) established on an inverted microscope (Nikon, TE2000U, Tokyo, Japan) was used to monitor the dynamic process of the end-to-end length of DNA. The detail of setup was as described before [20,36]. Briefly, a cover glass slide was glued on a glass slide to serve as the sidewall to anchor the DNA molecules. The glass slide was then glued with a structure made of PMMA to form a flow chamber, with one side to introduce the solution and the other side to outflow the solution using a syringe pump. The sidewall of the cover glass was coated with antidigoxygenin to link the dig end of the DNA. Then, the DNA-bead constructs were loaded into the cell to form a sidewall-DNA-bead structure. A permanent magnet controlled by a micromanipulator system (MP-285, Sutter Instruments, Novato, CA, USA) was used to exert force on the paramagnetic bead, so as to stretch the DNA. The movement of the paramagnetic bead was recorded by a CCD camera in real time. The Chamber was positioned on a platform made of an aluminum alloy.

Various concentrations of FeCl_3_ solution are mixed with TRIS solution (10 mM, pH = 7.2); then, an equal volume of DNA solutions is added for measurement of magnetic tweezers. The solution was incubated for 30 min at least at room temperature and introduced into the flow cell by using a syringe pump. In a typical measurement, we move the magnet from some distance to some close position to a paramagnetic bead; thus, a magnetic force is applied on the suspending bead. When a fixed magnetic force was executed to the bead, we monitored the end-to-end length of DNA in real-time to measure its conformational change.

## 3. Results and Discussion

**Figure 2:**
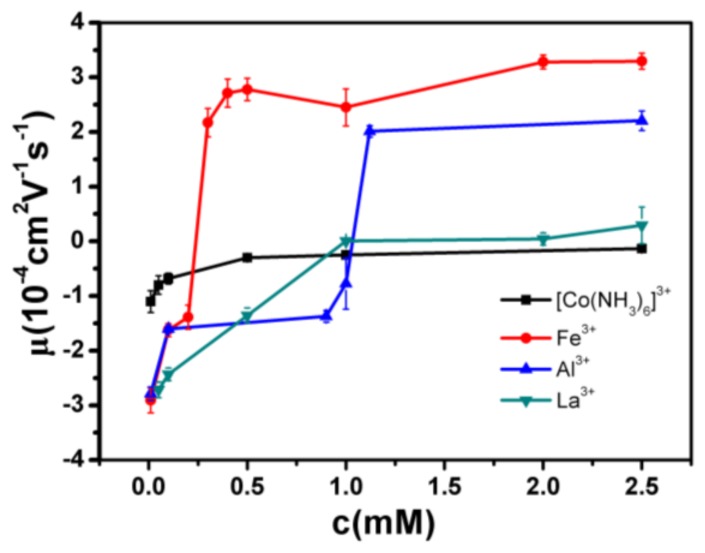
Electrophoretic mobility of DNA as a function of the concentration of different trivalent metal ions.

**Figure 3:**
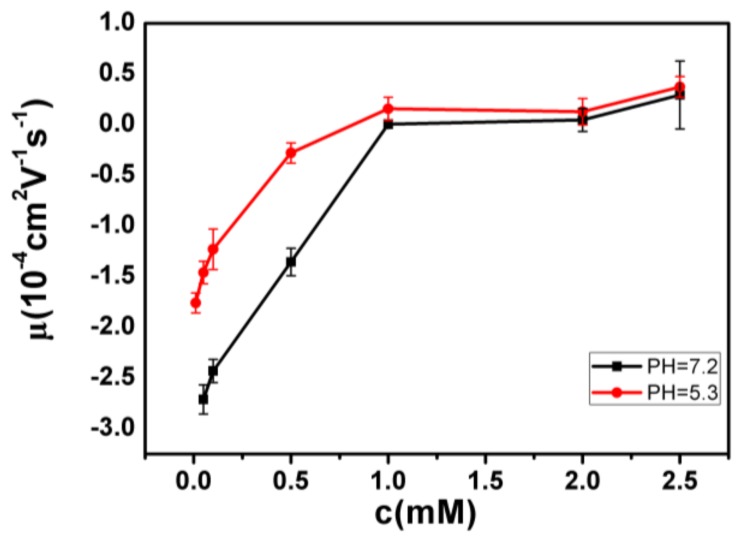
Electrophoretic mobility of DNA as a function of the concentration of different La^3+^ ions.

The measured electrokinetic properties of DNA in varous trivalent cations ion solution are shown in Figure 2, in which the electrophoretic mobility (EM) of DNA is plotted versus the concentrations of counterions. Two species of counterion with the same valance were used for consistency: Fe^3+^ and Al^3+^ for hydrolysed trivalent ions and La^3+^ and Co^3+^ for classical trivalent ions. We can see that the electrophoretic mobility of DNA goes up with the increase of concentration of cations. In classical La^3+^ and Co^3+^ aqueous solution, DNA generally does not invert its charge in the range of experimentally accessible concentration, as shown in Figure 2. At the highest La^3+^ concentration (2.5 mM), the charged DNA appears almost neutral; its electrophoretic mobility is around zero and sometimes slightly crosses the point. The mobility of DNA by Co^3+^ shows a similar tendency, but it never crosses the zero point, implying no charge inversion. However, in the aqueous solution of hydrolysed Fe^3+^ and Al^3+^, the scenarios of electrokinetics of DNA are changed drastically though the same valence of two species of cations. As can be seen from Figure 2, when the mixed solution of Fe^3+^ and DNA is 0.21–0.22 mM, the charge is suddenly reversed, and the Co^3+^ and La^3+^ are always at 0 value or less. In the aqueous solution of Al^3+^, charge inversion of DNA occurs around 1–1.1 mM. The transition width is quite narrow, 0.01 and 0.1 mM, respectively. As shown in Figure 3, we did the control experiment to observe the electrophoretic mobility of DNA as a function of the concentration of different La^3+^ ions. The results that are different from the charge inversion should be observed by using Fe^3+^ (and also Al^3+^) ions at pH from 7.1 to 5.3. For consistency, each data point is the average of five consecutive measurements, with the corresponding standard deviation as the error.

The transition of DNA is also reflected by its particle size measured by dynamic light scattering, which relates the spatial distribution of scattered light of particles in solution to their physical sizes. When the DNA is not agglomerated, the DNA is in a bulk linear group shape, and the convolution radius of the DNA is large, so that the particle size can be relatively large. If the DNA is condensed, so that a compact bulk structure can be formed, compared with a loose coil structure, the hydrodynamic radius can be reduced, so that the measured particle size is relatively small. We measured the particle size of λ-DNA and Fe^3+^, Al^3+^, Co^3+^, and La^3+^ mixture, respectively, by dynamic light scattering. 

**Figure 4:**
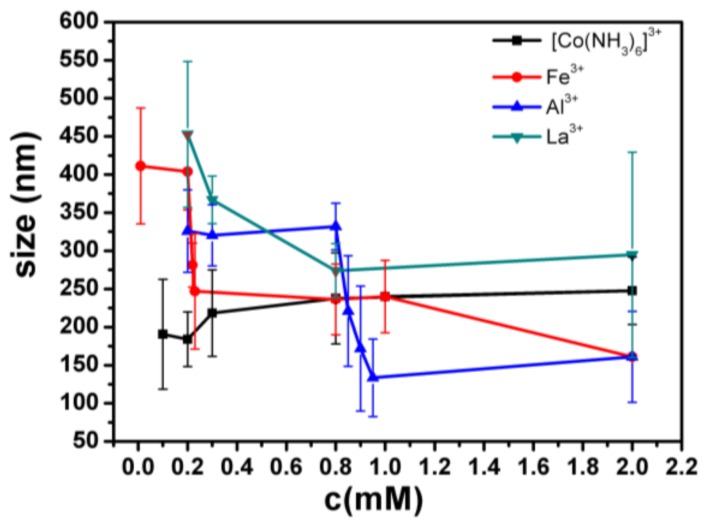
The size of DNA as a function of the concentration of different trivalent metal ions. The error of particle sizes is standard deviation, obtained from the CONTIN analysis software from DLS. In the measurement of particle size, there is only one peak at all different conditions for most cases, but two peaks in some fewer cases (<5%). We used only the one peak data for spread calculation.

In Figure 4, the hydrodynamic radius of the DNA molecules is represented as a function of the surfactant concentration, with data taken from the size distribution calculations As shown in Figure 4, the phenomenon that the particle size is suddenly reduced in the Fe^3+^ solution is obviously seen; the concentration of the reducing ions is about 0.2–0.3 mM, and the width of the reducing ions is 0.1; also, the particle size is suddenly reduced in the Al^3+^ solution, the concentration of the reducing ions is approximately 0.8–1 mM, and the width of the reducing ions is 0.2; in the La^3+^ solution, the particle size becomes smaller, the concentration of the reducing ions is approximately 0.2–1 mM, and the width of the reducing ions is 0.8; in the Co^3+^ solution, the particle size is basically kept unchanged.

**Figure 5:**
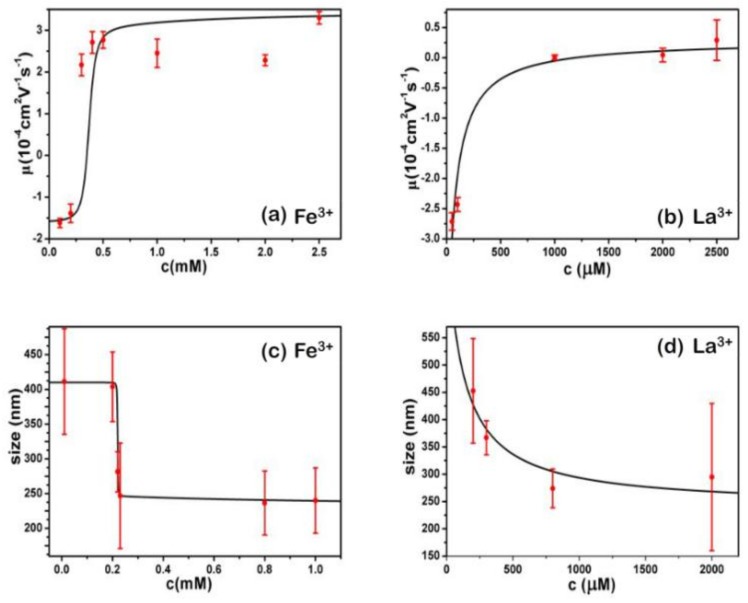
Electrophoretic mobilities and particle sizes of DNA-complex by Fe^3+^ (**a**,**c**) and La^3+^ (**b**,**d**) fitted by cooperative Zimm–Brag and simple two-state model, respectively.

We can see that both the electrophoretic mobility and particle size of DNA complex by hydrolysed metal ions show steep but continuous variation with the concentrations. They show the all-or-none nature of the transition in DNA chains, suggesting that the DNA compaction is highly cooperative. Thus, we apply a simple cooperative Zimm–Bragg model [37] to fit the experimental data, shown in Figure 5a,c for Fe^3+,^ respectively. 

In the case of Fe^3+^, the cooperative parameter σ is about 1 × 10^−4^, and σ is 1 × 10^−3^ for Al^3+^. The parameters are universal for curves of electrophoretic mobilities and particle size, implying consistency between charge neutralization and comformational change of DNA.

However, the electrophoretic mobility and particle size of DNA by neutral La^3+^ and Co^3+^ do not fit the cooperative Zimm–Bragg model instead of non-cooperative 2-state model [38]. The results’ fittings are shown in Figure 5 for electrophoretic mobility and particle size by La^3+^, respectively. The case of Co^3+^ is similar. The corresponding binding energy Δε = −9.5 K_B_T for La^3+^, Δε = −9.4 K_B_T for Co^3+^, respectively. The very close binding energies indicate the interaction between DNA and neutral counterions is purely electrostatic, being dependent only on the valence of ions and independent of the species of ions. 

The differences in DNA charge neutralization and condensation between hydrolysed and classical cations can be explained by their different DNA binding of the multivalent counterions and pH regulation. The capillary electrophoresis and the spectroscopic results for the Fe(III) complexes with DNA in aqueous solution showed that Fe(III) binds to the backbone phosphate group at low cation concentration, while at higher Fe(III) content, there are chelations via major groove and the phosphate group and no major perturbations of the base pairs, while Fe(III) causes DNA condensation, and no DNA conformational changes occurred upon Fe(III) complex formation, and DNA remains in the B-form [28,29,30,31].

As we have seen that the electrophoretic mobility and particle size of DNA by La^3+^ fit the simple two-state model of statistic mechanics, their binding to phosphates of DNA can be described by the extended Poisson Boltzmann theory treating DNA/ions system in an electrostatic model for the collective behavior of the anions (phosphate groups) at the interface. It has been shown that Fe (III) is mainly combined with the phosphate group of DNA; the cation directly acts on the phosphoric acid group on the skeleton, and the surface charge of DNA be neutralized, so the DNA charges can be reversed [39,40,41,42,43]. From the electrophoretic mobility curve in Figure 5a, we can see that under low-concentration trivalent acidic metal ions, the surface charge of the DNA molecule is basically kept stable, and when the concentration increases, the DNA charge is rapidly changed from a negative value to a positive value in a very narrow range.

The hydrolysis of metal ions, such as Fe^3+^ and Al^3+^, varies the solution pH due to their association to hydroxyls and release of protons. The limited solubility of hydroxide of metal ions leads to the decrease of pH in solution, which in turn promotes the charge neutralization of DNA because of the protonation of phosphates. The conditions (i.e., pH) under which the metal ion will hydrolyse are dependent on properties of metal ions. In general, the larger the charge and the smaller the ion radius, the lower the pH at which the metal will hydrolyse.

We focus on the effects of the trivalent metal ions Al^3+^, Fe^3+^, La^3+^, and Co^3+^ and, in particular, the effects of pH variations arising from the metal hydrolysis. We measured their pH values at various concentrations and found that pH value in the cases of La^3+^ and Co^3+^ vary little, while the values in the cases of Fe^3+^ and Al^3+^ decrease significantly due to the hydrolysis. For example, in the range of 0.1–1 mM of Fe^3+^, the pH of solution varies from 7.1 to 5.3, promoting slightly the protonation of phosphates to be favourable for charge inversion. Since the pKa of the phosphate groups in the phosphodiester linkage is around 1, we can expect only small change in the effective charge reducing the pH from 7.1 to 5.3 [44]. Thus, the promotion is insignificant.

**Figure 6:**
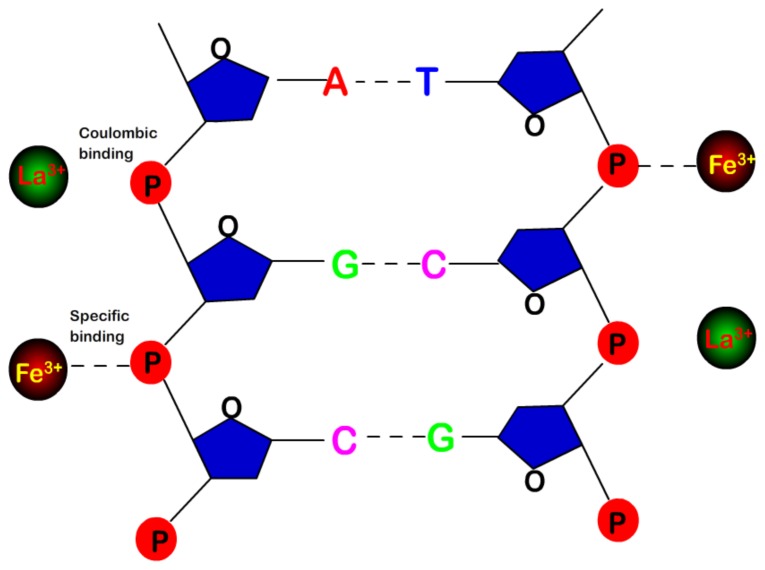
Schematic representation of the bindings of Fe^3+^ and La^3+^ to phosphates of DNA backbone, where the Fe^3+^ binding depends on its character in the solution and is highly specific, similar to covalent bond, while La^3+^ binding is electrostatic and sensitive to surface charge.

The mechanism of DNA charge inversion and compaction is schematically represented in Figure 6. Both neutral and hydrolysed metal ions, such as Fe^3+^ and La^3+^, bind to phosphates of DNA backbone. However, the Fe^3+^ binding depends on its character in the solution and is highly specific, similar to covalent bond, while La^3+^ binding is electrostatic and non-specific, being sensitive to DNA surface charge. In the meanwhile, the pH value of solution of acidic metal ions is reduced due to hydrolysis, which in turn might be slightly favorable for charge inversion and condensation of DNA. The neutral metal ions do not have the additional features.

As pointing out in Ref. [31,41], the binding of Fe to phosphate groups can be kept even when pH drops below 2, in which phosphate groups can be partially charged or totally neutralized. Thus, Fe(III) binding is not be a result of electrostatic interactions alone. Electrostatic interactions account for the initial attraction between Fe-ions and phosphate groups, and by virtue of the proximity to the phosphate groups and quantum effects, the Fe–phosphate can combine by bonding and likely be covalent. 

### 3.1. AFM Morphology of DNA

**Figure 7:**
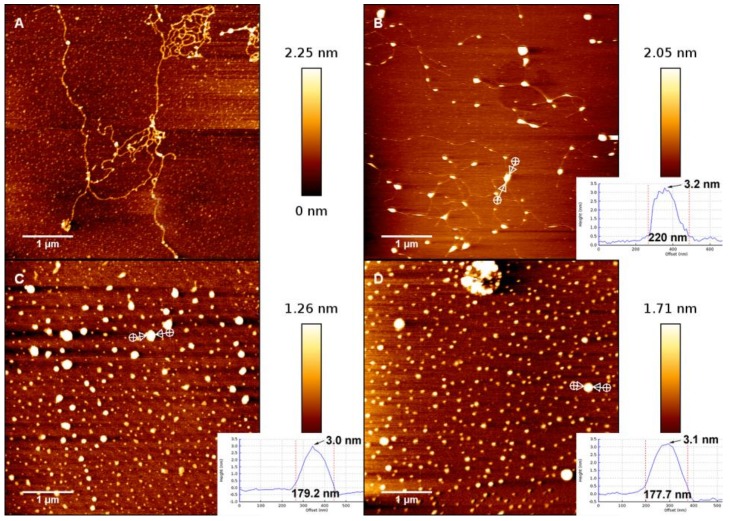
AFM observation of DNA complexes. Panel (**A**): λ-DNA in 0.0007 mM Fe^3+^ solution. Panel (**B**–**D**): DNA conformations at different concentrations (0.0009, 0.003, and 0.03 mM) of Fe^3+^ solution.

In order to further analyse the interaction between DNA and hydrolysed metal ions, we have imaged the change in the morphology of DNA-Fe^3+^ complex by means of AFM. Using the method described in the materials and methods section, a drop of Fe^3+^ and DNA mixture was deposited for 3 min on a freshly cleaved mica surface and then rinsed and dried for AFM imaging. The morphologies of DNA complexes in the presence of different concentration of Fe^3+^ in solution are shown in Figure 7A, which shows the morphology of DNA in absence of trivalent cations but in the same buffer condition as used for the complexes. We can see that the DNA molecules are well separated and show relaxed coils on the surface. When the concentration of Fe^3+^ in solution is increased and crosses over some critical concentration (about 0.8 μM), the condensation grows very rapidly. As shown in Figure 7B, in which the concentration of Fe^3+^ is 0.9 μM, we can see the compact nonspherical globules but still with some connecting DNA segments. These nonspherical globules can be considered as the intermediate states of the process of DNA condensation induced by hydrolysed Fe^3+^. When the concentration of iron ions grows further, the connecting DNA segments disappear; instead, spherical globules are the common compact structures, as in Figure 7C. In the case of high concentration, such as in Figure 7D, the spherical globules become more compact, corresponding to the smaller size of DNA particles. We measured the dimensions and heights of the dots to distinguish the collapsed DNA and salt crystals. We found that the height of collapsed DNA is about 3.3 nm, as shown in Figure 7B, in which a DNA thread connecting to the dot can be seen. In Figure 7C,D, the heights of DNA dots are unvaried, but the sizes decrease with increasing ion concentration, implying more compact structure of collapsed DNA. The smaller dots correspond to salt crystals, with heights of around 0.7 nm.

**Figure 8:**
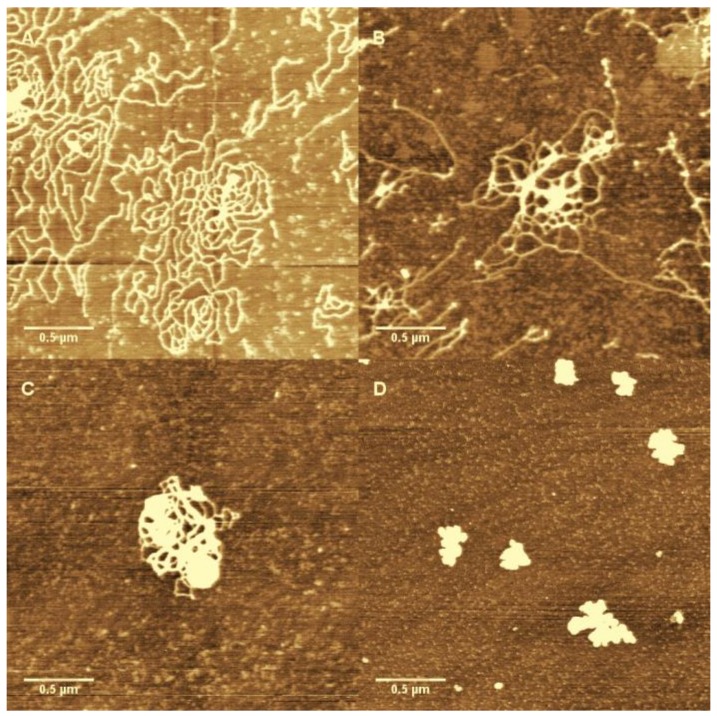
The AFM images of DNA condensation induce by Co^3+^. The concentration of Co^3+^ is 0.01 mM (**A**); 0.06 mM (**B**); 0.15 mM (**C**); and 0.2 mM (**D**).

We can clearly see that DNA changes from coiled to globular as ferric ion concentration increases. As we observed in the measurements of electrophoretic mobility and particle size by DLS, the direct visualization of DNA complex also shows the conformational transition in a very narrow width of concentration of Fe^3+^ ions. A notable feature of the DNA condensates is its compact globular structure, and there are almost no other condensing structures such as toroids and rods that appear in the case of classical counterions like Co^3+^ and spermine. We present the AFM images of DNA morphologies induced by Co^3+^ for comparison, shown in Figure 8, in which DNA condensates go through different intermediate sates to the final compact structure. We can see that the coil–globule transition by ferric ion is markedly discrete at the level of individual DNA molecules, i.e., the transition is all or none.

DNA morpgologies by hydrolysed metal ions show the simple coil–globule transition. However, some proteins like chromatin structural protein CTCFT are able to induce DNA to form more complex structure such as compact circular complexes, meshes, and networks [44].

### 3.2. Force Spectroscopy of DNA Complexes

**Figure 9:**
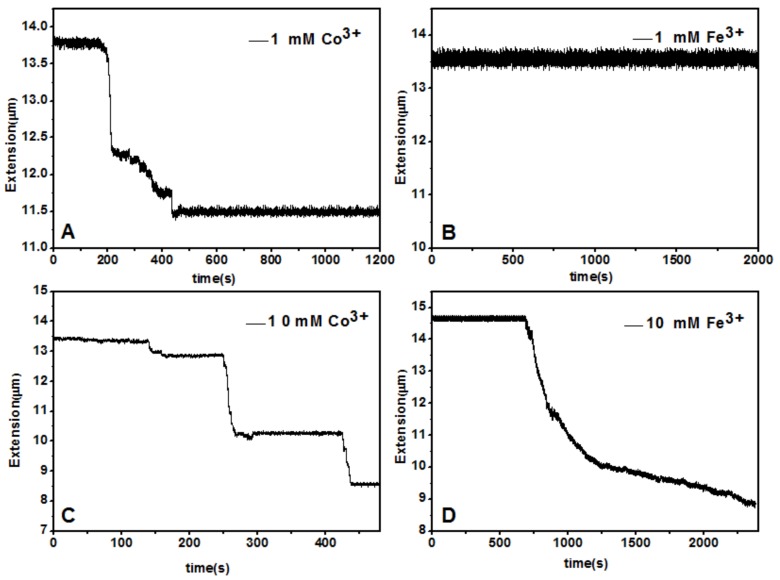
DNA extension-time curves at concentrations of Fe^3+^ (**b**,**d**) and Co^3+^ (**a**,**c**).

A magnetic tweezers (MT) setup was used to tether a single λ-DNA molecule (≈16.4 μm) by pulling the DNA-connected paramagnetic bead in TRIS buffer (10 mM, pH = 7.2) at room temperature (≈25 °C). After flowing couterion solution of various concentration into the sample cell, we can see the tethered DNA compaction and measure the applied force simultaneously. In the experiment, about 150 μL Fe^3+^ or Co^3+^ solution was loaded into the cell in each measurement. Condensing force is defined as the applied magnetic force to the bead when contractions in DNA extension-time curve occurred. The typical pulling curves of tethered DNA are presented in Figure 9, and the comparison between hydrolysed Fe^3+^ and La^3+^ shows their quite different properties. When concentration of cations is low, such as 1 mM, the pulling curve of Co^3+^ shows some distinct stepwise contraction in the condition of some applied force. In the case of Figure 9, the condensing force is 5.29 pN, a quite big value. In contrast, the condensing force induced by Fe^3+^ at the same concentration is almost negligible, less than 0.1 pN. In the condition of high concentration of counterions, such as 10 mM, the condensing force of Co^3+^ is about 4.11 pN, slightly less than that at low concentration. The decrease of condensing force can be attributed to charge inversion of DNA at high concentration of neutral Co^3+^. However, at the same concentration of Fe^3+^, DNA shrunk gradually rather than distinct stepwisely when a force F = 3.82 pN was applied to the bead.

From the observation, we can infer that DNA condensation induced by Co^3+^, as we have known [19], forms some higher structure like toroid or rod, while the DNA complexes by hydrolysed Fe^3+^ are simply globules, consistent with the images observed by AFM in last section.

It is notable that the measured critical concentration of condensation is different depending on the technique employed, since some additional interactions are involved in the measurement. In DLS, measurement is achieved in the bulk solution and no additional interactions are involved. In AFM imaging, the pretreated surfaces of mica are positively charged, and they significantly promote the condensation of DNA, implying the lower measured critical concentration. As for MT experiment, free DNAs in sample cell solution are washed away, and only the single DNAs linking the side wall are used for measurement, implying the DNA concentration in cell is much lower than the initial value. Additionally, in the measurement, DNA chain is pulled by a force, so the concentration of condensation is much larger than the value in DLS and AFM.

## 4. Conclusions

The main results of present study are as follows:

(1) Despite the fact that Fe^3+^ or Al^3+^, and La^3+^ or Co^3+^, have the same valence, they result in significantly different effects upon DNA in solution. Both kinds of trivalent counterions can lead to DNA compaction; the easily hydrolysed metal ions are able to invert the charge of DNA, while neutral La^3+^ and Co^3+^ are not.
(2) The two kinds of trivalent counterions behave distinctly differently in the charge neutralization and compaction of DNA. La^3+^ or Co^3+^ provide reasonable representations of “classical” ions, whose interaction with DNA can be described by a simple two-state model in statistical mechanics. In contrast, the hydrolysed Fe^3+^ and Al^3+^ show ionic specific interactions with phosphate groups of DNA. We used a cooperative Zimm–Bragg model to explain the observed conformational and electrophoretic mobility transition of DNA.
(3) DNA condensing force by hydrolysed Fe^3+^ and Al^3+^ is weaker than those by neutral La^3+^ and Co^3+^, and there are no stepwise jumps, but there is continuous shrinking in the pulling curves. Due to hydrolysis of Fe^3+^ and Al^3+^, pH variation not only regulates DNA charge but also plays a role in binding of the ions to DNA.

As in the case of charged interfaces [31], ion-specific interaction also has a significant influence on DNA compaction or condensation, as well as its charge neutralization and related charge inversion.
